# Intensified thermal management for patients undergoing transcatheter aortic valve implantation (TAVI)

**DOI:** 10.1186/1749-8090-6-117

**Published:** 2011-09-25

**Authors:** Ivo F Brandes, Marc Jipp, Aron F Popov, Ralf Seipelt, Michael Quintel, Anselm Bräuer

**Affiliations:** 1 Department of Anesthesiology, Emergency and Intensive Care Medicine, University of Göttingen, Robert-Koch-Str. 40, 37075 Göttingen, Germany; 2 Department of Thoracic and Cardiovascular Surgery, University of Göttingen, Robert-Koch-Str. 40, 37075 Göttingen, Germany

**Keywords:** Transcatheter aortic valve implantation, hypothermia, thermal management, core temperature, prewarming, forced air warming

## Abstract

**Background:**

Transcatheter aortic valve implantation via the transapical approach (TAVI-TA) without cardiopulmonary bypass (CPB) is a minimally invasive alternative to open-heart valve replacement. Despite minimal exposure and extensive draping perioperative hypothermia still remains a problem.

**Methods:**

In this observational study, we compared the effects of two methods of thermal management on the perioperative course of core temperature. The methods were standard thermal management (STM) with a circulating hot water blanket under the patient, forced-air warming with a lower body blanket and warmed infused fluids, and an intensified thermal management (ITM) with additional prewarming using forced-air in the pre-operative holding area on the awake patient.

**Results:**

Nineteen patients received STM and 20 were treated with ITM. On ICU admission, ITM-patients had a higher core temperature (36.4 ± 0.7°C vs. 35.5 ± 0.9°C, p = 0.001), required less time to achieve normothermia (median (IQR) in min: 0 (0-15) vs. 150 (0-300), p = 0.003) and a shorter period of ventilatory support (median (IQR) in min: 0 (0-0) vs. 246 (0-451), p = 0.001).

**Conclusion:**

ITM during TAVI-TA reduces the incidence of hypothermia and allows for faster recovery with less need of ventilatory support.

## Background

Aortic valve replacement with cardiopulmonary bypass (CPB) is currently the treatment of choice for symptomatic aortic stenosis but carries a significant risk of morbidity and mortality, particularly in frail elderly patients with severe comorbidities [1]. Transcatheter aortic valve implantation via the transapical approach (TAVI-TA) without CPB is a promising alternative in selected patients [2,3] but is associated with a high risk of perioperative hypothermia with several adverse side effects [4-7]. Hypothermia can be avoided by conductive warming methods [8,9] or forced-air warming [9-11]. Forced-air warming is an accepted method for preventing hypothermia in surgical patients [12] because of its well documented efficacy, [13-15] low costs, and ease of use. However, forced-air warming alone is not sufficient to prevent hypothermia for every operative procedure, [16-18] especially when it is used without prewarming [19]. Therefore, we compared prewarming with forced-air to no prewarming in patients undergoing TAVI-TA.

## Methods

After approval of our institutional review board we compared two methods of thermal management during TAVI-TA and their effects on the course of core temperature and time of postoperative ventilatory assist in this exploratory, observational study.

Patients were premedicated with a benzodiazepine, and had a balanced anesthesia with sevoflurane (1.0-1.2 MAC) and sufentanil. The trachea was intubated and ventilation was set to give normal end-tidal CO_2_. Inotropes and vasopressors were administered intraoperatively to maintain stable hemodynamics, if required. Patients were defined to be hemodynamically stable if their blood pressure was ± 15% of the initial blood pressure, if they developed no tachycardia (heart rate ≤ 90 bpm), and needed only moderate inotropes or vasopressors.

After the procedure, patients were transferred to the intensive care unit (ICU). They were extubated in the operating room (OR) if hemodynamically stable, and core temperature was above 35.5°C. If these criteria were not met, they remained intubated and ventilated, and were rewarmed and weaned from the ventilator in the ICU using our standard criteria for extubation (paO_2 _> 100 mmHg at FiO_2 _= 0.4, PEEP 5 mmHg, bladder temperature ≥ 35.5°C, patient hemodynamically stable).

Initial core temperature was taken with an infrared tympanic thermometer on the awake patient before induction of anesthesia. Intra- and postoperative core temperature was monitored with a thermistor-tipped Foley catheter after induction of anesthesia and recorded. Normothermia was defined as core temperature ≥ 36.0°C.

Standard thermal management (STM) consisted of an intraoperatively circulating hot water blanket under the patient, intraoperatively forced-air warming with a lower body blanket and warmed infused fluids.

The results of the first 19 patients managed with the standard method (STM) were considered clinically inadequate in regard to the thermal management. An intensified thermal management (ITM) was therefore implemented and a further 20 patients were measured. In ITM, initial core temperature was taken with an infrared tympanic thermometer before active warming with forced-air of the awake patient was started. Active warming was then started and continued throughout the induction phase of anesthesia. The time from the start of prewarming to scrubbing was 27 ± 18 min. During the operation we used a circulating hot water blanket under the patient, forced-air warming with a lower body blanket and warmed infused fluids.

Endpoints of the study were incidence of core temperature below 36.0°C, temperature at end of procedure, eligibility for extubation in the OR, and duration of mechanical ventilation.

After testing for normal distribution with Shapiro-Wilks test, data were analyzed with Student's *t *test, Mann-Whitney-U-test or repeated measure analysis of variance (ANOVA) with post hoc test, as appropriate. Categorical data were analyzed with Fisher's exact test. All normally distributed data are given as mean ± standard deviation. Not normally distributed data are given as median and interquartile range (IQR). A p < 0.05 was considered statistically significant.

A planned follow-up, prospective, randomized comparison was not given approval due to the *prima facie *superiority of the intensified thermal management regimen shown in our data.

## Results

**Figure 1:**
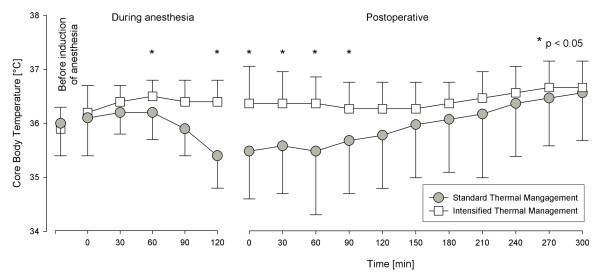
**Core body temperature before induction of anesthesia, during anesthesia, and during the first 300 min after admission to ICU**.

Demographic data and scores did not differ between the two groups (Table 1). There was no significant difference in the initial core temperature before induction of anesthesia (STM 36.0 ± 0.6°C vs. ITM 35.9 ± 0.4°C; p = 0.66), but ITM-patients had a higher core temperature before scrubbing (STM 36.2 ± 0.6°C vs. 36.6 ± 0.3°C; p = 0.008). Length of scrubbing and draping time were similar in both groups (STM 36.4 ± 12.5 min vs. ITM 36.4 ± 13.4 min; p = 0.99). Procedure time did not differ between both groups (STM 80 ± 21 min vs. ITM 74 ± 16 min; p = 0.329). ITM-patients had a significantly higher core temperature 60 and 120 minutes after induction of anesthesia and during the procedure (figure 1). On ICU admission, ITM-patients had a significantly higher core temperature (36.4 ± 0.7°C) compared to STM-patients (35.5 ± 0.9°C; p = 0.001). The incidence of hypothermia upon ICU admission was significantly higher in the STM group (13/19 vs. 5/20, p = 0.0077). These patients also needed longer to recover from hypothermia (median, IQR): STM 150 (0-300) min vs. ITM 0 (0-15) min, p = 0.003.

**Table 1:** Demographics and results

	STM	ITM	p
Number of patients	19	20	
Age, yrs (SD)	84 (3)	82 (6)	0.248
Height, cm (SD)	164 (7)	163 (8)	0.881
Weight, kg (SD)	75 (15)	71 (15)	0.431
Male, n (%)	4 (21)	5 (25)	1.000
Body surface area, m^2 ^(SD)	1.8 (0.2)	1.8 (0.2)	0.454
BMI, kg*m^-2 ^(SD)	27.9 (5.2)	26.7 (5.3)	0.632
EURO Score, % (SD)	26.6 (9.0)	26.6 (14.1)	0.993
Procedure time, min (SD)	80 (21)	74 (16)	0.329
Duration of prewarming, min (median; (IQR))	none	25; (15-32.5)	
Temperature before anesthesia or prewarming, °C (SD)	36.0 (0.6)	35.9 (0.4)	0.66
Temperature at begin of scrubbing, °C (SD)	36.2 (0.6)	36.6 (0.3)	0.008
Scrubbing time, min (SD)	36.4 (12.5)	36.4 (13.4)	0.99
Temperature at start of surgery, °C (SD)	36.0 (0.6)	35.9 (0.4)	0.66
Temperature at end of procedure, °C (SD)	35.6 (0.7)	36.4 (0.5)	0.001
Temperature at ICU admission, °C (SD)	35.5 (0.9)	36.4 (0.7)	0.001
Time until normothermia, min (median; (IQR))	150; (0-300)	0; (0-15)	0.003
Temperature afterdrop, °C (median; (IQR))	0.1; (0-0.4)	0.16; (0.05-0.5)	0.383
Ventilatory assist, hrs (median; (IQR))	4.1; (0-7.52)	0; (0-0)	0.001
Incidence of hypothermia, n (%)	13 (68)	5 (25)	0.0077
Extubation in OR, n (%)	6 (32)	18 (90)	0.0002

All normally distributed data are given as mean and standard deviation (SD), for not normally distributed data median and interquartile range (IQR) are given.

In the STM group, 13 of 19 patients could not be extubated in the OR because core temperature was below 35.5°C. In the ITM group, 18 of 20 patients could be extubated in the OR (p = 0.0002). The STM-patients also needed longer mechanical ventilation on the ICU (median, IQR): STM 4.1 (0-7.5) h vs. ITM 0 (0-0) h, p = 0.001.

## Discussion

Aortic valve surgery due to aortic stenosis is one of the most common cardiac procedures and an increasing number of patients with severe comorbidities are treated with transcatheter aortic valve implantation via the transapical approach (TAVI-TA) to avoid the use of cardiopulmonary bypass (CPB). During off-pump coronary artery bypass surgery (OPCAB) maintaining normothermia is challenging, as the absence of CPB also removes the opportunity to rewarm the patient on bypass [20]. This is also true for TAVI-TA.

Hypothermia after cardiac surgery is associated with coagulopathy, increased blood loss and more transfusions of packed red blood cells [7]. It is also associated with a higher release of troponin [6], prolonged mechanical ventilation, ICU and hospital length of stay and a significantly greater mortality [7,21].

In this study standard thermal management using intraoperatively a circulating hot water blanket under the patient, forced-air warming with a lower body blanket and warmed infused fluids was insufficient to maintain normothermia. Instead we observed a drop in core temperature throughout anesthesia and surgery.

Hypothermia is common during anesthesia and surgery. Practically all anesthetics and narcotics affect thermoregulation and therefore induction of anesthesia leads to redistribution of heat from the warm core of the body to the colder periphery [22,23]. Without active warming measures core temperature drops in a characteristic pattern in a cold operating room. During the first hour after induction of anesthesia redistribution of heat causes an initial large drop in core temperature. During the following 3 hours core temperature linearly decreases slower due to heat loss exceeding metabolic heat production and then core temperature stops dropping [23].

Even with sufficient active intraoperative warming measures the drop of core temperature due to redistribution of heat can be observed and core temperature starts to rise again between 20 minutes to 3 hours after induction of anesthesia [8-10,15,16]. Our result of a dropping core temperature during surgery is therefore in agreement with the data given in the literature.

In contrast to the STM-patients the ITM-patients using prewarming combined with consequent intraoperative warming had a reduced incidence and degree of hypothermia. The efficacy of prewarming has been shown in several clinical studies [10,19]. However, this result is remarkable, because several studies using forced-air warming during OPCAB surgery have failed to demonstrate efficacy, although in some of these studies patients were also actively prewarmed [5,24-27]. Therefore, several authors recommend very expensive thermal management methods like water garments [6,24] or adhesive water mattresses [4,28].

This difference between OPCAB surgery and TAVI-TA surgery can be explained by the fact that during OPCAB surgery large areas of the body surface are exposed to ambient room temperature during surgical skin preparation and during the procedure. Normally, both legs are exposed for vein harvesting and the thorax is opened via a sternotomy. Therefore only special cardiac surgical forced-air warming blankets can be used and these blankets cover only a very small area of the body. In contrast, during TAVI-TA less body surface is exposed and more area is left for forced-air warming. Both legs, one groin, and the right part of the thorax can be covered with forced-air warming blankets. The fact that the skin under a forced-air warming blanket is no longer an important source of heat loss [29] but a source of heat gain, changes the heat balance of the body and is responsible for the efficacy of forced-air warming.

## Conclusions

In conclusion, patients undergoing TAVI-TA benefit from an intensified perioperative thermal management. They are less likely to become hypothermic, have a higher core temperature on ICU admission, recover faster from hypothermia, and need less mechanical ventilation. In contrast to patients undergoing OPCAB, prewarming and consequent intraoperative warming with forced-air is sufficient in patients with TAVI-TA to avoid perioperative hypothermia, and there is no need to use very expensive measures to keep these patients normothermic.

## Competing interests

RS is proctor for Edwards Lifesciences.

AB has acted as consultant for LMA Deutschland GmbH and 3 M Deutschland GmbH.

Authors IFB, MJ, AFP, and MQ do not have any competing interests.

## Authors' contributions

IFB participated in designing the study, carried out the experimental work, data analysis, statistical evaluation, and drafted the manuscript. MJ participated in designing the study, and carried out the experimental work. AFP participated in the data analysis and preparation of the manuscript. RS performed the surgeries and participated in the manuscript preparation. MQ participated in the manuscript preparation. AB participated in designing the study, data analysis, and participated in the manuscript preparation. All authors read and approved the manuscript.
